# Fenfluramine and Comparative Antiseizure Therapies in Drug-Resistant Epilepsy: A Systematic Review of Efficacy, Cardiometabolic Safety, and Clinical Outcomes

**DOI:** 10.7759/cureus.91949

**Published:** 2025-09-10

**Authors:** Ashis Pal, Bhavna Singla, Shivam Singla, Muhammad Habib Ur Rehman, Ali Hamza, Ahmad Mohammad, Momina Abid, Aqib Imran

**Affiliations:** 1 Internal Medicine, Dhaka National Medical Institute Hospital, Dhaka, BGD; 2 Internal Medicine, Erie County Medical Center Hospital, Buffalo, USA; 3 Internal Medicine, TidalHealth Peninsula Regional, Salisbury, USA; 4 Internal Medicine, King Edward Medical University, Lahore, PAK; 5 Internal Medicine, Hurley Medical Center, Flint, USA; 6 Internal Medicine, Madinah Teaching Hospital, Faisalabad, PAK; 7 Internal Medicine, Nishtar Medical University, Multan, PAK

**Keywords:** antiseizure medications, cardiovascular safety, dravet syndrome, epilepsy, fenfluramine, lennox-gastaut syndrome, long-term efficacy, metabolic outcomes, pediatric neurology, seizure control

## Abstract

Fenfluramine has emerged as a promising adjunctive antiseizure medication for treatment-resistant epilepsy syndromes such as Dravet and Lennox-Gastaut. This systematic review synthesized evidence from seven studies, including randomized controlled trials and open-label extensions, evaluating fenfluramine’s efficacy, cardiovascular and metabolic safety, and long-term outcomes across pediatric and adult populations. The findings consistently demonstrated substantial reductions in seizure frequency, particularly for drop and generalized tonic-clonic seizures. Safety monitoring revealed no cases of valvular heart disease or pulmonary arterial hypertension, and treatment-emergent adverse events such as decreased appetite and fatigue were generally mild to moderate. In parallel, selected studies evaluating other commonly used antiseizure medications provided comparative insights into metabolic risks, identifying concerns such as vitamin D deficiency, hyponatremia, and weight fluctuations, alongside mitigation strategies through supplementation and lifestyle interventions. Integrating these data contextualizes fenfluramine’s favorable profile within the broader antiseizure therapy landscape and underscores the importance of individualized risk-benefit assessment. Overall, the evidence supports fenfluramine as an effective and well-tolerated option for drug-resistant epilepsy, while emphasizing the need for systematic metabolic monitoring to optimize long-term care. Future research should prioritize head-to-head comparative trials, standardized cardiovascular and metabolic safety protocols, and long-term real-world studies to strengthen clinical guidance and advance precision therapy in epilepsy management.

## Introduction and background

Epilepsy is a chronic neurological disorder characterized by recurrent, unprovoked seizures, affecting approximately 50 million people globally [1]. The mainstay of treatment involves the long-term use of antiepileptic drugs (AEDs), also known as antiseizure medications (ASMs), which are essential for seizure control and improving quality of life [2]. While these medications have transformed epilepsy management, concerns remain regarding their long-term cardiovascular and metabolic safety [3,4].

Over the past two decades, research has increasingly highlighted the extraneurological effects of AEDs. Older agents such as valproate, carbamazepine, and phenytoin have been linked to dyslipidemia, weight gain, insulin resistance, and elevated cardiovascular risk [5,6]. Although newer AEDs like levetiracetam, perampanel, and eslicarbazepine are generally considered safer, emerging evidence suggests that their long-term cardiometabolic effects also warrant closer evaluation.

Because patients with epilepsy often require lifelong therapy, cumulative adverse effects can be significant, particularly in vulnerable groups such as children, the elderly, and those with comorbidities like obesity, diabetes, or hypertension. The metabolic burden of AEDs may further compound the already high risk of cardiovascular disease (CVD), which remains a leading cause of morbidity and mortality worldwide [7]. Yet despite its clinical importance, much of the literature remains narrowly focused on efficacy and seizure reduction, with limited attention to cardiovascular and metabolic endpoints [8].

Fenfluramine, recently approved for Dravet and Lennox-Gastaut syndromes, has emerged as a promising adjunctive ASM with expanding clinical use. Given its prior association with cardiotoxicity when used as an anorectic agent, its reintroduction in epilepsy care underscores the need for rigorous evaluation of both efficacy and long-term safety. This systematic review therefore synthesizes current evidence on fenfluramine, while also drawing on comparative findings from other ASMs to contextualize its cardiometabolic profile. By emphasizing both efficacy and safety, the review aims to inform safer prescribing practices and guide individualized monitoring strategies in drug-resistant epilepsy.

## Review

Materials and methods

Study Design and Reporting Framework

This systematic review was conducted and reported in accordance with the Preferred Reporting Items for Systematic Reviews and Meta-Analyses (PRISMA) 2020 guidelines [9]. Although the protocol was not prospectively registered, it was developed in adherence to standardized systematic review methodology. The PICO (Population, Intervention, Comparator, Outcome) framework [10] guided the research question and study selection. The population included pediatric and adult patients with drug-resistant epilepsy, particularly those with Dravet syndrome or Lennox-Gastaut syndrome. The primary intervention of interest was fenfluramine (FFA) as adjunctive therapy, with comparators including placebo or other antiseizure medications (ASMs). The primary outcome was reduction in seizure frequency, while secondary outcomes included cardiovascular safety (e.g., valvular heart disease, pulmonary arterial hypertension) and metabolic effects (e.g., appetite changes, weight, and biochemical markers).

Search Strategy

A comprehensive literature search was conducted across PubMed, Scopus, and Cochrane Controlled Register of Trials (CENTRAL) to identify studies evaluating fenfluramine and, where relevant, other ASMs with reported cardiometabolic outcomes. Search terms included “fenfluramine,” “Dravet syndrome,” “Lennox-Gastaut syndrome,” “drug-resistant epilepsy,” “antiepileptic drugs,” “antiseizure medications,” “cardiovascular safety,” and “metabolic effects.” Boolean operators and Medical Subject Headings (MeSH) were applied to optimize search sensitivity. The search was limited to English-language publications from the past 10 years. Reference lists of included articles were screened manually to identify additional eligible studies.

Eligibility Criteria

Studies were eligible if they examined fenfluramine in drug-resistant epilepsy and reported at least one of the following: seizure frequency outcomes, cardiovascular safety, or metabolic effects. In addition, studies evaluating cardiometabolic safety of other commonly prescribed ASMs were included when they provided contextual relevance for interpreting fenfluramine’s safety profile. Eligible designs included randomized controlled trials (RCTs), open-label extension (OLE) studies, and observational studies with clearly reported clinical or safety outcomes. Exclusion criteria were pediatric epilepsy studies that did not assess fenfluramine, case reports, editorials, narrative reviews, non-English publications, and studies lacking full-text access or relevant outcome data.

Study Selection

Two reviewers independently screened the titles and abstracts of all retrieved articles. Full texts were then reviewed to determine eligibility based on the predefined criteria. Discrepancies were resolved through discussion, with a third reviewer consulted as needed. Ultimately, seven studies met the inclusion criteria. A PRISMA flow diagram summarizing the study selection process is available in the supplementary materials.

Data Extraction and Synthesis

A standardized data extraction form was used to collect study details, including design, sample size and population characteristics, fenfluramine dosage and regimen, comparator treatments, follow-up duration, seizure outcomes, and any reported cardiovascular or metabolic findings. Fenfluramine studies formed the core synthesis, focusing on efficacy, cardiovascular safety, and metabolic outcomes. In addition, selected studies of other antiseizure medications and adjunctive interventions were extracted to provide comparative context on cardiometabolic safety. Due to heterogeneity in study design, outcome definitions, and intervention protocols, all findings were synthesized narratively, and a meta-analysis was not performed.

Risk of Bias Assessment

The Cochrane Risk of Bias 2.0 (RoB 2) tool [11] was applied to randomized controlled trials, while the Risk Of Bias In Non-randomized Studies - of Interventions (ROBINS-I) tool [12] was used for non-randomized studies. Each study was evaluated across domains including selection bias, performance bias, detection bias, attrition, and reporting bias. Two reviewers independently assessed risk of bias, with disagreements resolved by consensus. Final assessments were categorized as low, moderate, or serious risk and presented in tabulated form for transparency.

Results

Study Selection Process

**Figure 1:**
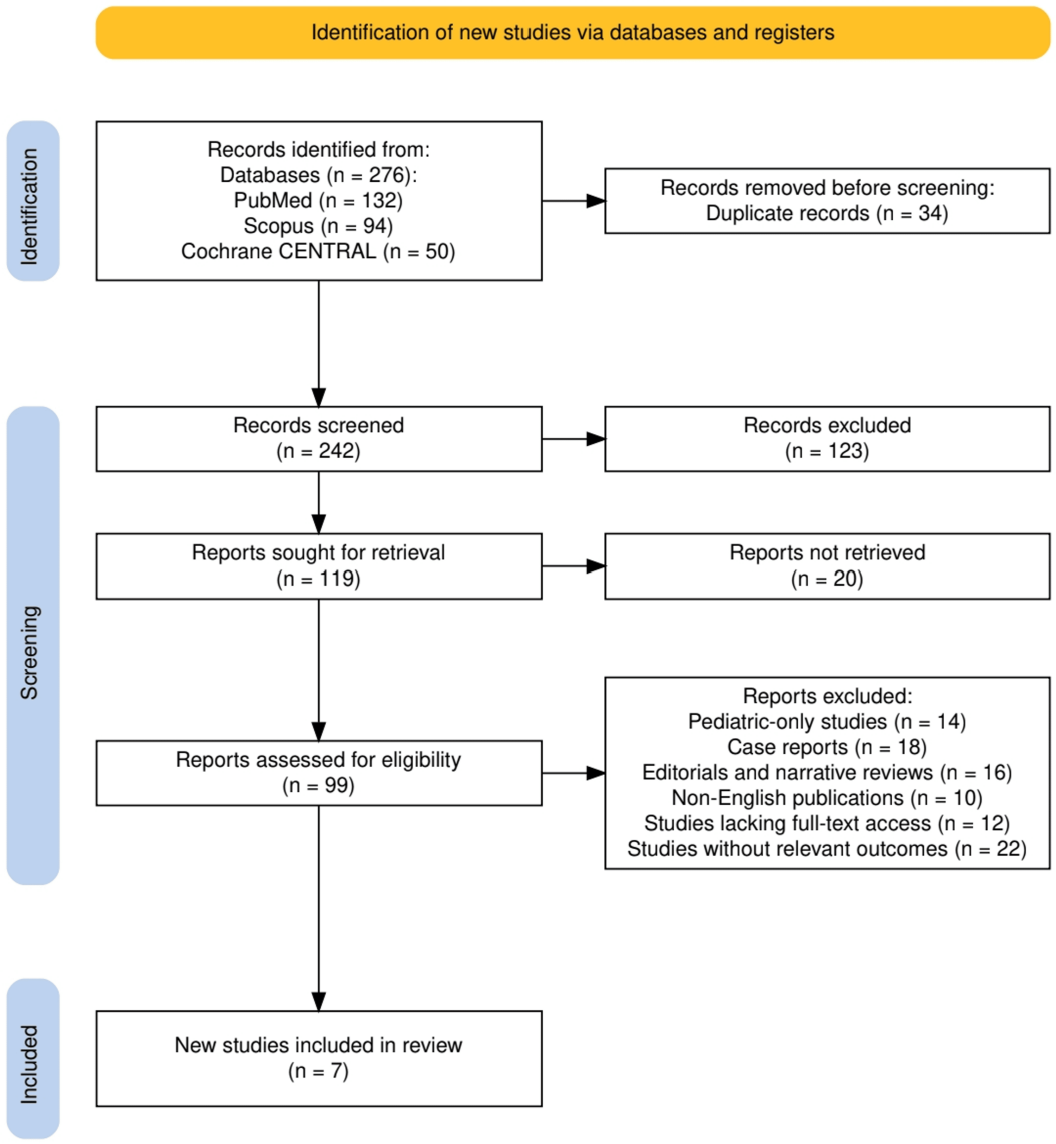
The PRISMA flow diagram represents the study selection process. PRISMA: Preferred Reporting Items for Systematic reviews and Meta-Analyses

Figure 1 illustrates the systematic process of study selection for this review. An initial total of 276 records were identified across three databases: PubMed (n=132), Scopus (n=94), and Cochrane CENTRAL (n=50). After removing 34 duplicates, 242 records were screened for relevance. Of these, 123 were excluded based on title and abstract screening, while 119 full-text articles were assessed for eligibility. Ultimately, 92 reports were excluded due to predefined criteria such as pediatric-only focus, case reports, editorials, narrative reviews, non-English language, lack of full-text access, or absence of relevant outcomes. This rigorous process resulted in the inclusion of seven studies in the final review.

Characteristics of the Selected Studies

Table 1 summarizes the key characteristics of the seven studies included in this review. The majority of studies were randomized controlled trials or open-label extensions, enrolling both pediatric and adult populations with drug-resistant epilepsy, particularly Dravet syndrome and Lennox-Gastaut syndrome. Fenfluramine was the most frequently assessed therapy, with studies consistently demonstrating significant reductions in seizure frequency, including drop seizures and generalized tonic-clonic seizures. Importantly, no major cardiovascular adverse events, such as valvular heart disease or pulmonary arterial hypertension, were reported across long-term follow-up. Treatment-emergent effects such as decreased appetite and fatigue were generally mild to moderate in severity. In addition, selected studies of other ASMs and non-pharmacological adjuncts were included to provide comparative context on cardiometabolic safety. Oxcarbazepine and eslicarbazepine acetate, for example, were associated with metabolic side effects such as hyponatremia, particularly in pediatric and elderly populations. Adjunctive measures, including sodium supplementation and home-based exercise regimens, were also evaluated for their impact on metabolic parameters and physical functioning. Overall, while fenfluramine demonstrated a favorable balance of efficacy and safety, findings from contextual studies highlight that long-term metabolic and cardiovascular risks remain an important consideration across the broader ASM landscape.

**Table 1:** The characteristics of the selected studies in the systematic review. RCT: Randomized controlled trial; OLE: open-label extension; TEAE(s): treatment-emergent adverse event(s); ADR: adverse drug reaction; PWE: people with epilepsy; ASM(s): anti-seizure medication(s); AED(s): anti-epileptic drug(s); CGI-I: Clinical Global Impression – Improvement; GTCS: generalized tonic-clonic seizures; BMI: body mass index; PCS: Physical Component Score; VHD: valvular heart disease; PAH: pulmonary arterial hypertension; 25-OHD: 25-hydroxyvitamin D; ALP: alkaline phosphatase; iPTH: intact parathyroid hormone; Ca/Cr ratio: calcium to creatinine ratio; HDL: high-density lipoprotein; LDL: low-density lipoprotein; FFA: fenfluramine; ESL: eslicarbazepine acetate; 6MWT: 6-minute walk test.

Study (Author, Year)	Study Design	Population (n, age group)	AED(s) Used	Comparator	Duration of Follow-up	Cardiovascular Outcomes	Metabolic Outcomes	Main Findings
Knupp et al. (2022) [13]	Randomized Controlled Trial	263 patients with Lennox-Gastaut syndrome, aged 2–35 years (median 13 years)	Fenfluramine (0.2 or 0.7 mg/kg/day)	Placebo	14 weeks (2-week titration+12-week maintenance)	No cases of valvular heart disease or pulmonary arterial hypertension observed	Decreased appetite reported in 22%	Fenfluramine significantly reduced drop seizure frequency, especially at 0.7 mg/kg/day. Greater response seen in generalized tonic-clonic seizures. Common adverse effects included decreased appetite (22%), somnolence (13%), and fatigue (13%). No serious cardiovascular events were noted.
Scheffer et al. (2025) [14]	Open-Label Extension Study (after RCTs)	374 patients with Dravet Syndrome (including 45 adults aged 19–35); median exposure 824 days	Fenfluramine (FFA)	None (OLE)	Up to 3.5 years	No valvular heart disease or pulmonary arterial hypertension. Abnormal echocardiography observed only as physiological regurgitation.	Decreased appetite and blood glucose among TEAEs	FFA showed a 66.8% median reduction in monthly convulsive seizure frequency (MCSF). Adults had ~70% improvement on CGI-I scale. Patients without concomitant stiripentol had greater MCSF reduction than those with it. No new or unexpected safety signals. Findings confirm sustained efficacy and long-term safety.
Knupp et al. (2023) [15]	Open-Label Extension Study (Post RCT)	247 patients with Lennox-Gastaut syndrome; mean age 14.3±7.6 years (32% adults); 88.3% on 2-4 ASMs	Fenfluramine (FFA)	None (OLE)	Median 364 days (12+ months)	No cases of valvular heart disease (VHD) or pulmonary arterial hypertension (PAH) observed	Decreased appetite (16.2%), fatigue (13.4%)	Median drop seizure frequency reduced by 28.6% (entire OLE), and by 50.5% at month 15. Non-drop seizures reduced by 45.9%. GTCS and tonic seizures had the most reduction (48.8% and 35.8%, respectively). CGI-I ratings: ~36% rated as "Much/Very Much Improved." FFA well tolerated; no serious long-term cardiopulmonary complications.
Vichutavate et al. (2022) [16]	Randomized Controlled Trial	82 children and adolescents (5–18 years) on AEDs >6 mo	Various AEDs (not specified)	High-dose vs. standard-dose ergocalciferol	90 days	Not assessed	Serum 25-OHD, calcium, phosphate, magnesium, ALP, iPTH, urine Ca/Cr ratio	High-dose ergocalciferol significantly improved vitamin D status; 80.5% normalized vs. 36.6% with standard dose. Higher BMI Z-score linked to persistent deficiency.
Neha et al. (2024) [17]	Randomized Controlled Trial	120 children (1–18 years) with epilepsy	Oxcarbazepine (monotherapy)	Sodium chloride (1–2 g/day) vs. no supplementation	12 weeks	Not assessed	Serum sodium, urine sodium, serum/urine osmolality, incidence of hyponatremia	Sodium chloride reduced incidence of hyponatremia (4/60 vs. 14/60, p=0.01). No significant effect on symptomatic/severe hyponatremia, behavior, cognition, or seizures.
Sudhindra Vooturi et al. (2020) [18]	Randomized Controlled Trial	110 PWE (mean age 25.85±9.62 years); 55 per group	Various AEDs (not specified)	Home-based exercise vs. control	12 weeks	6MWT distance improved in exercise group (464.29 m vs. 293.07 m, p=0.007)	Weight loss (−7.65 kg vs. +4.01 kg, p<0.001); improved HDL, reduced LDL in obese PWE	Exercise group had significant weight reduction, better 6MWT performance, and improved PCS score. AED type/number did not influence outcomes. No seizures reported during follow-up.
Magalhães et al. (2021) [19]	RCT + Post-marketing review	1983 adults (120 elderly ≥65 y; 1863 non-elderly)	Eslicarbazepine acetate (ESL)	Elderly vs. non-elderly	Up to 8 years (post-marketing)	Not directly assessed	Hyponatremia more frequent in elderly (6.7% vs. 1.5%); most reported ADR in elderly (14.6%)	No new safety signals identified. Elderly had more serious TEAEs, possibly due to comorbidities. Safety profile of ESL in elderly comparable to non-elderly after 8 years.

Risk of Bias Assessment

As shown in Table 2, the risk of bias across the included studies ranged from low to moderate, depending on study design and methodological rigor. Randomized controlled trials generally demonstrated low risk when appropriate randomization, blinding, and clear outcome reporting were employed. However, some trials raised concerns due to unclear blinding or reliance on subjective or self-reported outcomes. Open-label extensions and observational studies, evaluated using ROBINS-I, were judged to have a moderate risk of bias due to their non-randomized nature, lack of comparators, and potential for confounding, although consistent safety monitoring and transparent reporting improved their overall reliability.

**Table 2:** The risk of bias assessment of all the included studies.

Study (Author, Year)	Study Design	Tool Used	Risk of Bias Judgment	Key Comments
Knupp et al. (2022) [13]	Randomized Controlled Trial	RoB 2	Low	Well-randomized, blinded design with minimal missing outcome data.
Scheffer et al. (2025) [14]	Open-Label Extension	ROBINS-I	Moderate	Non-randomized open-label design; no comparator; consistent safety monitoring, but risk of confounding.
Knupp et al. (2023) [15]	Open-Label Extension	ROBINS-I	Moderate	No control group and unblinded design; outcome reporting transparent but some selection bias possible.
Vichutavate et al. (2022) [16]	Randomized Controlled Trial	RoB 2	Some concerns	Blinding unclear; randomization method not fully described; objective outcomes reduce bias risk.
Neha et al. (2024) [17]	Randomized Controlled Trial	RoB 2	Low	Adequate randomization and allocation; outcomes clearly defined and well reported.
Vooturi et al. (2020) [18]	Randomized Controlled Trial	RoB 2	Some concerns	Non-blinded intervention (exercise); self-reported metabolic outcomes may introduce measurement bias.
Magalhães et al. (2021) [19]	Observational/Post-marketing	ROBINS-I	Moderate	Non-randomized subgroup comparison; potential confounding by age and comorbidities handled appropriately.

Discussion

Across the included studies, fenfluramine demonstrated substantial efficacy in reducing seizure frequency in patients with Lennox-Gastaut syndrome and Dravet syndrome, particularly for generalized tonic-clonic and drop seizures. This benefit is thought to arise from fenfluramine’s unique serotonergic and sigma-1 receptor modulation, mechanisms that likely broaden its anticonvulsant activity beyond that of many traditional antiepileptic drugs (AEDs) [20]. Despite historical concerns regarding fenfluramine’s cardiotoxicity when used as a weight loss agent, none of the recent epilepsy trials - including long-term extensions of up to 3.5 years - reported clinically significant valvular heart disease or pulmonary arterial hypertension, supporting a favorable risk-benefit profile at therapeutic dosages [21]. Appetite suppression was a consistent finding, reflecting its serotonergic action, but was generally mild and may even represent a therapeutic advantage in patients with obesity-related comorbidities. Importantly, the reassuring cardiovascular outcomes observed in these trials likely reflect not only the drug’s safety at low doses but also rigorous patient selection, structured echocardiographic monitoring, and careful exclusion of those with preexisting cardiac disease.

Comparative insights from other AEDs help contextualize fenfluramine’s cardiometabolic profile. Older-generation agents such as valproate and phenytoin have long been associated with adverse effects, including weight gain, lipid derangements, and insulin resistance [22]. By contrast, fenfluramine demonstrated a minimal cardiometabolic burden in the available studies, more closely aligning with the favorable safety profile reported for newer agents evaluated under strict trial conditions, such as those by Knupp et al. [13] and Scheffer et al. [14]. This contrast reinforces a broader paradigm shift in epilepsy management-from reliance on older AEDs with significant metabolic risks to evidence-based, individualized therapy with newer agents. Within this framework, fenfluramine emerges as a promising adjunctive treatment, combining robust seizure control with a manageable safety profile, provided that structured monitoring protocols remain in place.

The comparative pharmacology of other antiseizure medications further contextualizes fenfluramine’s role in drug-resistant epilepsy. Agents such as levetiracetam act by binding to synaptic vesicle glycoprotein 2A (SV2A), stabilizing neuronal firing and reducing excitatory neurotransmitter release, while benzodiazepines like diazepam enhance GABAergic (gamma-aminobutyric acid) inhibition, leading to broader central nervous system (CNS) suppression [23,24]. These mechanisms highlight the heterogeneity of ASM action and underscore why efficacy and tolerability profiles differ across agents.

However, many commonly used ASMs carry safety trade-offs. Older drugs such as phenytoin and benzodiazepines are frequently associated with sedation, dizziness, and cognitive slowing, which can be particularly problematic in vulnerable groups such as children and the elderly. Levetiracetam is linked with behavioral changes, including irritability and aggression, while oxcarbazepine has been implicated in metabolic complications such as hyponatremia [25]. In contrast, fenfluramine demonstrated a favorable safety profile across included trials, with no major cardiovascular concerns and only mild to moderate appetite suppression or fatigue. These findings reinforce its potential as a safer adjunctive option compared to older-generation ASMs, provided that routine cardiovascular and metabolic monitoring is maintained.

Emerging evidence indicates that treatment responses and safety outcomes with ASMs can vary significantly across patient subgroups. Factors such as age, comorbidities, and seizure type influence both efficacy and tolerability, underscoring the importance of stratified treatment approaches rather than uniform prescribing [26]. For example, children and elderly patients may be more vulnerable to metabolic complications or behavioral side effects from certain ASMs, while individuals with comorbid cardiovascular or metabolic disease require closer monitoring for drug-related adverse effects. Within this landscape, fenfluramine has shown consistent seizure reduction with relatively few cardiometabolic concerns, suggesting its potential value in carefully selected populations.

The clinical implications of these findings emphasize the need for individualized, precision-based epilepsy management. Broad-spectrum application of older ASMs with known long-term metabolic or cardiovascular risks is increasingly difficult to justify, particularly when newer therapies such as fenfluramine or eslicarbazepine demonstrate more favorable safety profiles in well-monitored trials [27]. Future guideline development should prioritize stratified prescribing strategies, incorporate systematic metabolic and cardiovascular monitoring, and build on long-term extension data to optimize safety in vulnerable groups. These insights align with the broader move toward precision medicine in epilepsy care.

The available literature on fenfluramine and other ASMs in drug-resistant epilepsy primarily consists of randomized controlled trials (RCTs) and open-label extensions, supplemented by observational studies. While RCTs offer higher internal validity through randomization, blinding, and standardized endpoints, they are often limited by modest sample sizes and relatively short follow-up, restricting the ability to capture long-term cardiometabolic outcomes. Open-label and observational studies provide more real-world insights but remain susceptible to selection bias and confounding. Heterogeneity across studies - including differences in outcome definitions (e.g., seizure frequency vs. responder rates), duration of follow-up, and monitoring protocols - further complicates cross-trial comparisons and limits generalizability beyond the study populations. Additionally, most available data come from high-resource settings, raising concerns about applicability in low- and middle-income countries (LMICs), where patient demographics, comorbidities, and treatment availability may differ substantially.

Several important evidence gaps persist. Long-term assessments of cognitive function and quality of life remain scarce despite the recognized neuropsychiatric side effects of many ASMs. Cardiovascular and metabolic endpoints - highly relevant given the known arrhythmogenic and metabolic liabilities of older agents - are often underreported, leaving uncertainty about comparative safety in diverse populations. Head-to-head trials directly comparing fenfluramine with other commonly used ASMs are lacking, making it difficult to formulate agent-specific recommendations for clinical practice. Pediatric and elderly populations also remain underrepresented despite their unique pharmacokinetic, developmental, and comorbidity-related considerations. Finally, the absence of robust data from LMICs highlights an urgent need for globally inclusive studies to guide context-appropriate epilepsy management. Addressing these gaps will be essential for optimizing treatment strategies, improving long-term safety, and advancing precision medicine in epilepsy care.

## Conclusions

This systematic review highlights fenfluramine as an effective and generally well-tolerated adjunctive therapy for drug-resistant epilepsy, particularly in Dravet and Lennox-Gastaut syndromes. Across studies, fenfluramine consistently reduced seizure frequency, including generalized tonic-clonic and drop seizures, while demonstrating a favorable cardiovascular and metabolic safety profile when used with structured monitoring. Comparative findings from other ASMs underscore persistent concerns such as weight fluctuations, vitamin D deficiency, and hyponatremia, further contextualizing fenfluramine’s relative advantages. Nonetheless, evidence gaps remain regarding long-term cardiometabolic outcomes, cognitive effects, and quality of life, as well as limited data from pediatric, elderly, and LMIC populations. Future research should prioritize head-to-head comparative trials and standardized safety monitoring to refine precision therapy and optimize long-term care in drug-resistant epilepsy.
